# Acute neurocognitive and subjective effects of oral methamphetamine with low doses of alcohol: A randomised controlled trial

**DOI:** 10.1177/02698811231179805

**Published:** 2023-06-14

**Authors:** Amie C Hayley, Brook Shiferaw, Joanna Rositano, Luke A Downey

**Affiliations:** 1 Centre for Human Psychopharmacology, Swinburne University of Technology, Hawthorn, VIC, Australia; 2 Institute for Breathing and Sleep (IBAS), Austin Health, Melbourne, VIC, Australia; 3 International Council for Alcohol, Drugs and Traffic Safety (ICADTS); 4 Seeing Machines, Fyshwick, Canberra, ACT, Australia; 5 Forensic Science SA, Adelaide, SA, Australia

**Keywords:** Methamphetamine, alcohol, cognitive, combined, randomised controlled trial

## Abstract

**Background:**

Methamphetamine is often recreationally co-consumed with alcohol due to desirable off-target effects; however, the acute neurocognitive and subjective consequences of combined use are unclear.

**Methods:**

In a randomised, placebo-controlled, counterbalanced, cross-over study design, the effects of acute oral methamphetamine (0.42 mg/kg) were assessed with and without low doses of alcohol (target 0.04% blood–alcohol concentration, BAC) on subjective intoxication, alertness, physiological outcomes and neurocognition during the ascending and descending phases of the BAC curve. Sixteen healthy adults (mean age = 30.4 years, SD ± 4.4, 67% male) completed four experimental sessions over 4 weeks involving a one-week washout period.

**Results:**

Cardiovascular measures [heart rate (beats/minute), blood pressure (mmHg)] were predictably elevated following methamphetamine, but unaffected by combined alcohol use. Methamphetamine and alcohol produce divergent effects on subjective alertness and sedation across time, yet their combination produced predominantly sustained stimulative effects independent of the biphasic alcohol curve. At a peak BAC of 0.029%, alcohol alone impaired performance across most functional neurocognitive domains relative to placebo and methamphetamine only, and the addition of methamphetamine attenuated these effects. Methamphetamine alone produced isolated improvement in psychomotor speed consistent with peak drug effects.

**Conclusion:**

Methamphetamine combined with alcohol does not substantially alter the physiological or metabolic profile compared to either drug alone. Strong stimulative effects of methamphetamine appear to mask the biphasic sedative and performance effects of low doses of alcohol, which may underlie motivations for co-consumption in recreational settings and increase propensity for harm.

## Background

Methamphetamine, a potent central nervous system stimulant, is an indirect agonist at the noradrenaline, dopamine and serotonin (5-HT) receptor sites (Cruickshank & Dyer, 2009). It is one of the most common clandestinely and illegally manufactured, trafficked and abused amphetamine-type stimulant in the world, and its use is associated with increasing social, economic and health burden (United Nations, 2020). Despite this, explicit examination of methamphetamine-induced effects on pertinent behavioural and neurocognitive domains is limited. Of the few studies that are available, evidence of an inverted U-shaped concentration–effect relationship suggests that while low to moderate doses of methamphetamine may have no (Ermakova et al., 2014) or only select beneficial effects on functional neurocognition (Kirkpatrick et al., 2012b), these effects become maladaptive at higher doses and during tasks of greater complexity (Narayan et al., 2021). Recreationally, however, methamphetamine is rarely (if ever) consumed in isolation; yet even fewer studies have explored the relevant neurocognitive and subjective consequences of the comorbid use of methamphetamine with other substances such as alcohol.

Methamphetamine is frequently co-consumed with alcohol in recreational settings among occasional (Wu et al., 2007) and frequent stimulant users (Wang et al., 2017). Combined use may enhance desirable substance-specific properties and induce additive and/or synergistic pharmacological effects (Singh, 2019). Due to opposing mechanisms of action and mutually inflammatory properties, combined use can also amplify respective deleterious properties and increase propensity for toxicity (Singh, 2019). Over one-third of individuals admitted to an emergency department for trauma will have measurable concentrations of both methamphetamine and alcohol in their system (Isoardi et al., 2019), and the combined use of these substances is of increasing concern for road safety (Hayley et al., 2022). To our knowledge, only two small laboratory studies exist which examine the physiological and behavioural effects of combined methamphetamine and alcohol use. Under both acute (Mendelson et al., 1995) and repeated (Kirkpatrick et al., 2012a) protocols, individuals indicate higher self-reported ‘good drug effect’ relative to either substance alone, providing some evidence for potential *incentives* for their combined use; however, broader functional neurocognitive and relevant subjective effects (separate to craving) remain poorly defined. While acute methamphetamine administration reduces feelings of alcohol intoxication (Mendelson et al., 1995) and alcohol-induced performance deficits in a divided attention task (Kirkpatrick et al., 2012a), these studies are somewhat limited by the target populations (high frequency stimulant user cohorts) and use of fixed dosing paradigm, which may mask the magnitude of effects, particularly among other user groups.

This study aimed to examine the effects of oral methamphetamine alone and in combination with low doses of alcohol on subjective intoxication and neurocognitive performance in healthy volunteers with limited prior exposure to methamphetamine to better characterise potential interactions.

## Methods

### Design

This trial used a randomised, placebo-controlled, counterbalanced, double-blind, four-way within-subjects design with a minimum washout period of 1 week between study visits. Each participant received 0.42 mg/kg methamphetamine and vodka drink (with matched placebo capsule or drink) in a randomised order. Study visits occurred at the same time each week to control for any circadian variation in performance. Order of administration and treatment combination was determined via computer-generated randomisation software. Researchers conducting the study were blinded and were not involved in generating the randomisation schedule nor allocating participants to different drug conditions. The study was registered on the Australian New Zealand Clinical Trials Registry (ACTRN: 12618000629235) and was approved by the Swinburne University Human Research Ethics Committee (HREC: 20210770-8822).

#### Participants

A total of 21 participants aged between 21 and 40 years and who self-reported previous recreational use (lifetime history, less than 2 times/month) of amphetamine-type substances and who weighed less than 100 kg were recruited (Appendix 1). Individuals who reported current use of any medication for the treatment of a medical condition (except of oral contraceptives in women or treatment for benign conditions); who indicated a current or past diagnosis of physical, gastrointestinal, neurological or psychiatric condition, personal history of head-injuries or loss of consciousness (determined via self-report and confirmed in clinical interview) or who returned a blood pressure reading above 160/90 were excluded. Female participants were required to submit a negative pregnancy test at the start of each study visit.

#### Study treatments and analytical procedures

##### Methamphetamine

The active treatment was 0.42 mg/kg methamphetamine hydrochloride (purity 99.8 ± 1.3%). Weighted doses were rounded to the nearest whole number (e.g. weight of 74 kg × 0.42 mg = 31.08 mg, provided 30 mg). Raw product (batch numbers 14-D-12 and 19-D-01) was compounded into 5, 10 and 20 mg size #00 opaque capsules with microcrystalline cellulose filler by an independent compounding chemist. Identical matched placebo capsules comprised microcrystalline cellulose only. Methamphetamine oral fluid concentrations were measured at ~40 min and 3-h after consumption using the Quantisal Oral Fluid Collection Device (IMMUNALYSISTM).

Methamphetamine analysis was carried out by Forensic Science South Australia, per the method described in Rositano et al.’s study (2016) with some modification. All samples were analysed in duplicate, and the extraction automated on a Perkin Elmer Janus Liquid Handling Platform (Mulgrave, Victoria, Australia). Calibration curves were generated by spiking a 1:3 mixture of human oral fluid (Golden West Diagnostics, LLC) and oral fluid collection buffer (Statsure Diagnostic Systems Inc., Brooklyn, New York, United States) at 5–250 ng/mL methamphetamine (Cerilliant, Round Rock, Texas, United States) and D5-methamphetamine (Cerilliant, Round Rock, Texas, United States) internal standard spiked at a concentration of 50 ng/mL. Samples (100 µL) were diluted with aqueous ammonia (8%, 80 µL) and internal standard (20 µL, 250 ng/mL), then loaded onto the SLE sorbent (Biotage Isolute^®^ Uppsala, Sweden, SLE+200 96-well plates). After 5 min absorption the samples were eluted with methyl t-butyl ether (Sigma-Aldrich Macquarie Park, NSW, Australia, 900 µL) into a 96-well collection plate having glass inserts containing 50 µL aqueous formic acid (25%). The eluant was evaporated and reconstituted in methanol (80 µL, LiChrosolv^®^, Sigma Aldrich, Macquarie Park, NSW, Australia). Collection plates were capped and centrifuged (2000 rpm, 5 min) then transferred to a Shimadzu Nexera LC system [Shimadzu Scientific Instruments (Oceania) Pty. Ltd. Rydalmere, NSW, Australia] fitted with a Restek Ultrabiphenyl (Restek Corporation, Bellefonte, Pennsylvania, United States) (3 µm × 50 mm × 2.1 mm) column and Phenomenex (Torrance, California, United States) 2.1 mm PFP guard cartridge. The mobile phase was a gradient of acetonitrile (Optima LCMS grade, Fisher Scientific, Hampton, New Hampshire, United States) and 0.1% formic acid (Optima, Fisher Scientific, Hampton, New Hampshire, United States) over 6 min with a flowrate of 0.5 mL/min. LC/MS analysis was performed on a SCIEX QTrap 5500 mass spectrometer (Mt Waverley, Victoria, Australia) in positive ion mode with scheduled multiple reaction monitoring of three transitions m/z 150/119, m/z 150/91, m/z 150/65 with m/z 150/119 used for quantitation and confirmation of identification. Method validation included replicate spikes at 7.5, 125 and 300 ng/mL over 3 days giving overall inter- and intra-day accuracy of less than 15% (*n* = 63) and relative standard deviation of 4%. Calibration was linear (*r*^2^ > 0.999) with 1/x weighting without forcing through zero. The lower and upper limits of quantitation were 5 and 250 µg/L, respectively. Any samples above this range were diluted and reanalysed. Reported results are as determined from the buffer-diluted oral fluid samples.

##### Alcohol

The beverage was vodka (40% alcohol, Absolut brand) measured according to each participant’s body weight for a target blood–alcohol concentration (BAC) of 0.04% (0.38 g alcohol/kg body weight) mixed with 300 mL orange juice. The placebo condition consisted of orange juice with ~3 ml of vodka floated on top for masking.

Breathalyser readings were taken using a Lion Alcolmeter SD400PA that was calibrated quarterly by the Road Policing Division, Victoria Police. Approximation of BAC was determined though breath alcohol content at 20 min, 1 h and 3 h after treatment.

#### Measurement tools

##### Subjective measures

*Brief Biphasic Alcohol Effects Scale*: The Brief Biphasic Alcohol Effects Scale (B-BAES) is a validated efficient version of the BAES (Martin et al., 1993). Participants self-rate subjective feelings of being energised, excited and up (Stimulant subscale) and feelings of being sedated, slow thoughts and sluggishness (Sedation subscale) using a six-point Likert scale (Rueger and King, 2013). Scale scores are calculated as the sum of respective items. The B-BAES was measured at four timepoints to examine the biphasic stimulant and sedative effects of the treatment(s) over time and per condition.

*Karolinska Sleepiness Scale*: The Karolinska Sleepiness Scale (KSS) was used to assess psycho-physical state two times per session. Responses ranges from 1 (extremely alert) to 9 (very sleepy, great effort to keep awake, fighting sleep; Åkerstedt and Gillberg, 1990).

##### Neurocognitive performance

Functional neurocognitive performance was measured using the CogPro (Bristol, United Kingdom) and Cogtrack (Bristol, United Kingdom) online cognitive testing system(s) (Wesnes et al., 2017).

*Simple and choice reaction time*: The simple reaction time task measures speed at which a simple motor response is made to an expected stimulus occurring at repeated, unpredicted intervals. The choice reaction time task measures aptitude to focus concentration and process information. Outcomes included simple reaction time mean (in milliseconds, ms), choice reaction time mean (ms) and choice reaction time accuracy (%).

*Digit vigilance*: The 3-min Digit Vigilance task measures sustained and intensive attention (vigilance) by recording the number of correct detections (accuracy, %), as well as reaction time (ms) and error responses (False Alarms).

*Numeric working memory*: A target series of 5 digits (0 to 9) is presented one at a time on screen with each digit displayed for 1150 ms and a 50-ms interval between each presentation. Reaction time (ms) and accuracy of response to presented stimuli (%) were recorded.

*Spatial working memory*: A randomised presentation order of a series of ‘probe’ stimuli was shown on screen one at a time. Reaction time (ms) and accuracy (% accuracy to ‘original’ and ‘new’ stimuli) were recorded.

#### Procedure

**Figure 1.**
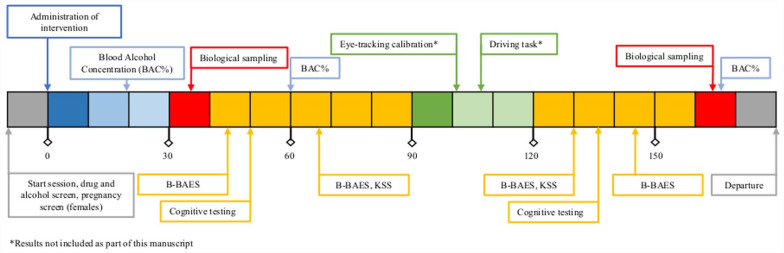
Overview of study session procedures.

On the first visit, eligibility was confirmed and participants who met inclusion criteria completed a training session on all performance tasks. Once completed, approval for inclusion was confirmed by the study physician, and participants were booked for their consecutive four-visit dosing protocol over 4 weeks. Figure 1 displays an overview of the study schedule on testing days. Upon arrival at the testing site for the dosing sessions, participants first underwent a breathalyser analysis to confirm absence of alcohol use, oral fluid screening to confirm absence of drug use (Drugwipe 6s screen; cannabis, opiates, cocaine, amphetamines/methamphetamines/ecstasy and benzodiazepines), and medication/lifestyle history was reviewed to assess ongoing eligibility. Alcohol and caffeine were prohibited for 12 h, and participants were instructed to have the same light meal no later than 2 h prior to each study visit to control for variability in gastric absorption of the study treatments (Vinarov et al., 2021). Participants were provided with a combination of capsules and a drink according to the scheduled treatment allocation. Approximately 30 min after dosing, participants completed the first B-BAES questionnaire and first cognitive assessment suite. The second B-BAES was completed at approximately 1-h post-dosing, along with the first KSS. The third B-BAES, second KSS and final cognitive suite were completed at ~2 h post-dosing, and the final B-BAES was completed at approximately 2.5 h post-dosing. Participants were prohibited from driving to/from the testing visit (and for 24-h post-testing) and were provided a taxi voucher upon site departure.

#### Analysis

A linear mixed model with restricted maximum likelihood estimation was used to examine the effects of condition (placebo, alcohol, methamphetamine, methamphetamine and alcohol), time and their interaction on neurocognitive and subjective outcomes. Using the likelihood-ratio statistic to determine the most parsimonious model, compound symmetry was considered best fit. Condition and time were entered into as the repeated factor, and separate models were built to examine each outcome. Where a main effect was observed, post-hoc paired t-tests with Bonferroni correction for multiple comparisons were conducted to examine condition and time differences. Considering the potential effect of sex differences in pharmacokinetics of alcohol and alcohol-induced dopamine release in healthy volunteers (Baraona et al., 2001), a bivariate analysis was performed on performance data as a function of sex. Where a significant effect was observed, sex was included as a co-variate in multivariable models. All statistical analyses were conducted with the use of SPSS 28.0 (SPSS Inc., USA), and all tests were two-tailed with a conventional level of significance of *p* < 0.05.

## Results

The quality of cognitive performance data was compromised for four participants due to technical problems (data loss due to COVID-19 pandemic shutdowns), and results from a further *n* = 1 participant was found to have violated protocol after unblinding. As such, analysis was conducted on the remaining 16 individuals (6 females, 10 males) who had complete data across the four experimental sessions.

### Demographic outcomes

Demographic, health and substance use characteristics are presented in Table 1. On average, participants were aged 30.4 years (SD ± 4.3), weighed 76.5 kg (range 56.9–99.0 kg) and had a body mass index of 25.1 kg/m^2^ (range 20.2–33.9 kg/m^2^). Participants predominantly self-reported their ethnicity as Caucasian (93.8%) and were, on average, highly educated. By design, all participants reported a lifetime history of amphetamine usage. All included participants reported prior alcohol or cannabis consumption (100%), and 81.3% reported having used cocaine at least once in their lifetime. No participants reported having ever used heroin.

**Table 1.** Demographic and lifestyle characteristics (*N* = 16).

Demographic parameters	Mean (±SD); *n*(%)
Sex, *n*(%)	
Male	10 (62.5)
Weight (kg) (±SD)	76.5 (13.5)
BMI (kg/m^2^) (±SD)	25.1 (4.1)
Age (years) (±SD)	30.4 (4.3)
Race/ethnicity, *n*(%)	
Caucasian	15 (93.8)
Other/mixed	1 (12.5)
Education level, *n*(%)	
Primary school	–
Secondary school	2 (12.5)
Some tertiary/diploma	8 (50.0)
Postgraduate	6 (37.5)
Employment status, *n*(%)	
Part-time/casual	9 (56.3)
Full-time employment	6 (37.5)
Unemployed	1 (6.3)
Drug use history, *n*(%)^a^	
Amphetamine	16 (100)
Alcohol	16 (100)
Cannabis/marijuana	16 (100)
Cocaine	13 (81.3)
Heroin	–

a Positive indication of ‘ever used’ only.

### Clinical outcomes

**Figure 2.**
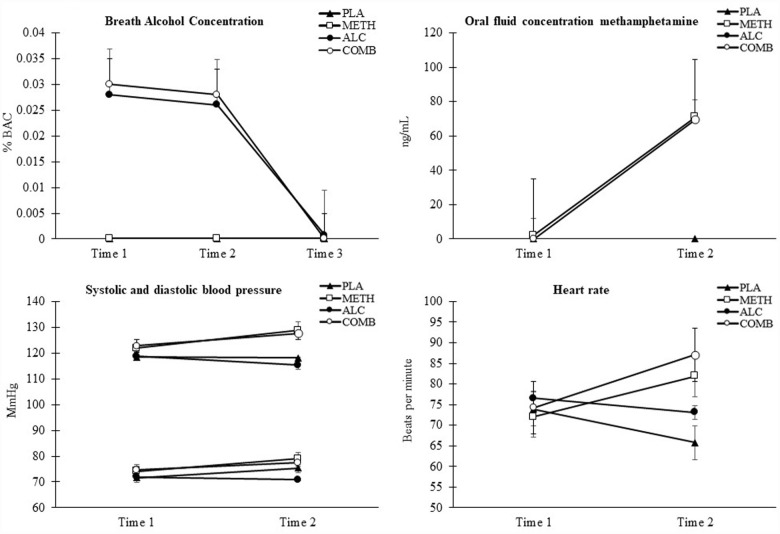
Upper panel (left): Breath alcohol concentration as a function of drug condition and time. Upper panel (right): Oral fluid concentration of methamphetamine (ng/ml) as a function of drug condition and time. Lower panel (left): Systolic and diastolic pressure (mmHg) as a function of drug condition and time. Lower panel (right): Heart rate (beats/minute) as a function of drug condition and time. Error bars represent standard error of measurement (±SEM). Overlapping error bars were omitted for clarity.

Breath alcohol and oral fluid methamphetamine concentrations (ng/mL) per condition and timepoint are presented in Figure 2, respectively (top panels). Methamphetamine concentrations for the methamphetamine and methamphetamine and alcohol condition(s), and BAC levels for the alcohol and methamphetamine and alcohol condition(s) were comparable at each collection timepoint (*p* *>* 0.05). Peak BAC was 0.029% for the alcohol condition and 0.030% BAC for the methamphetamine and alcohol condition, both reported at Time 1. Oral fluid concentrations of methamphetamine were comparable between the methamphetamine and methamphetamine and alcohol condition, respectively, 2.1 ng/ml (range 00–22 ng/mL) and 0.00 ng/ml (range 0.0–0.0 ng/mL) at Time 1 and 71 ng/ml (range 18–195 ng/mL) and 70 ng/ml (range 22–195.00 ng/mL) at Time 2.

#### Cardiovascular effects

Figure 2 (bottom two panels) displays cardiovascular outcomes as a function of drug condition and time. Overall, heart rate was elevated in the methamphetamine and alcohol condition relative to placebo (mean difference = 9.39, *p* = 0.002) and heart rate was increased in both the methamphetamine and methamphetamine and alcohol condition from Time 1 to Time 2 (mean difference = 8.31, *p* *=* 0.014; −13.70, *p* < 0.001, respectively). At Time 2, heart rate was elevated in the methamphetamine (mean difference = 11.93, *p* *=* 0.026) and methamphetamine and alcohol condition (mean difference = 19.39, *p* = 0.002) relative to both placebo, and for the methamphetamine and alcohol condition relative to alcohol (mean difference = 14.22, *p* = 0.003). Relative to alcohol and placebo, the methamphetamine and methamphetamine and alcohol condition increased systolic BP (all *p* < 0.05), and elevated diastolic BP relative to alcohol alone (all *p* *<* 0.05).

#### Subjective effects

**Figure 3.**
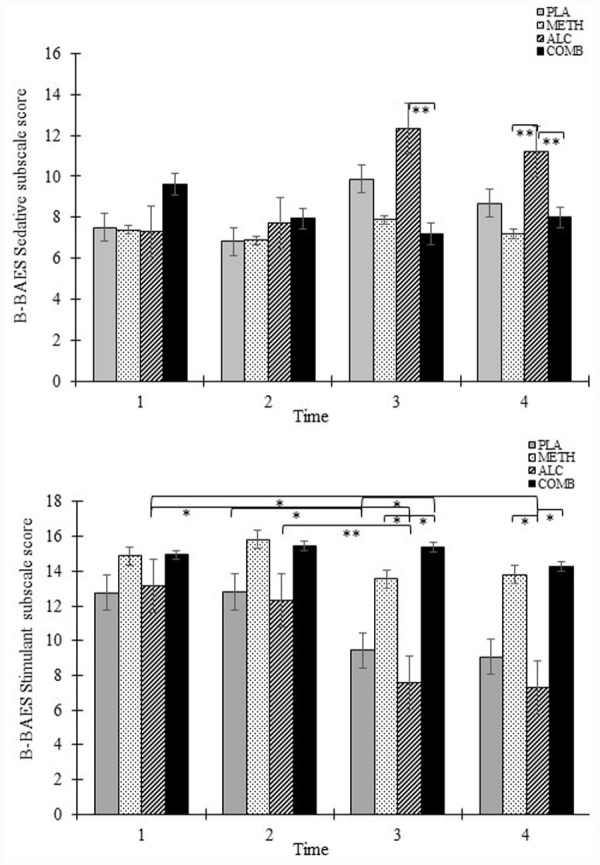
Upper panel: Brief Biphasic Alcohol Effect Scale (B-BAES) Sedative subscale score as a function of drug condition and time. Lower panel: B-BAES Stimulant subscale score as a function of drug condition and time. Error bars represent 1 SEM. Error bars represent ±SEM. *p *<* 0.05. **p < 0.001.

**Figure 4.**
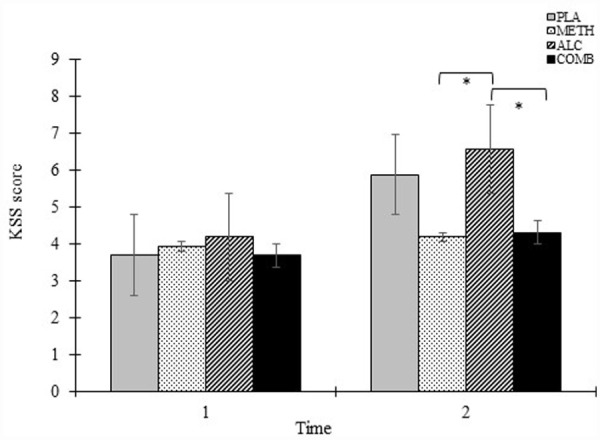
Karolinska Sleepiness Scale (KSS) score as a function of drug condition and time. Error bars represent 1 ± SEM. *p *<* 0.05. **p < 0.001.

A summary of B-BAES for the Stimulant and Sedation subscales is presented in Figure 3 and KSS results per condition and time are presented in Figure 4.

##### Brief Biphasic Alcohol Effects Scale

*Sedative effects*: The Sedation subscale of the B-BAES demonstrated a significant main effect of condition (F_(3, 225)_ = 4.49, *p* = 0.004) but not time (F_(3, 225)_ = 2.31, *p* = 0.077), and a significant interaction effect (F_(3, 225)_ = 2.41, *p* = 0.012). Alcohol produced a greater sedating effect relative to placebo (mean difference = 0.87, *p* = 0.18) and methamphetamine (mean difference = 0.97, *p* = 0.006) but not the methamphetamine and alcohol condition. Subjective sedation scores were comparable between conditions for Times 1 and 2, but significantly diverged at Time 3, with greater sedation in the alcohol relative to methamphetamine and alcohol condition (mean difference = 5.21, *p* = 0.024). There were differences in condition at Time 4, with greater sedation in the alcohol relative to methamphetamine (mean difference = 6.50, *p* = 0.004) and methamphetamine and alcohol condition (mean difference = 6.93, *p* = 0.002; See Figure 3 top panel).

*Stimulant effects*: The Stimulant subscale of the B-BAES demonstrated a significant main effect of condition (F_(3, 225)_ = 16.74, *p* < 0.001) and time (F_(3, 225)_ = 6.79, *p* < 0.001), but not its interaction (*p* > 0.05), with increased stimulant effects in the methamphetamine condition relative to the placebo (mean difference = 3.50, *p* < 0.001) and alcohol condition (mean difference = 4.44, *p* < 0.001) but not the methamphetamine and alcohol condition (*p* > 0.05). Stimulant effects were greater in the methamphetamine and alcohol condition relative to placebo (mean difference = 3.98, *p* < 0.001) and alcohol (mean difference = 4.92, *p* < 0.001). Stimulant scores decreased significantly over time in the alcohol (F_(3, 204)_ = 6.25, *p* < 0.001) and placebo condition (F_(3, 204)_ = 2.97, *p* = 0.033), but not in the methamphetamine or methamphetamine and alcohol condition. Subjective stimulant scores were comparable between conditions for Times 1 and 2, but significantly diverged at Times 3 and 4 (Figure 3 bottom panel).

##### Karolinska Sleepiness Scale

There was a significant main effect of condition (F_(3, 105)_ = 5.59, *p* = 0.005), time (F_(1, 105)_ = 19.96, *p* < 0.001), and its interaction (F_(3, 105)_ = 3.14, *p* = 0.028) on KSS score, with greater overall sleepiness in the alcohol condition relative to methamphetamine (mean difference = 1.31, *p* = .017) and methamphetamine and alcohol condition (mean difference = 1.37, *p* = 0.011; See Figure 3). KSS scores were comparable between conditions at Time 1 but significantly was greater at Time 2 in the alcohol condition relative to methamphetamine (mean difference = 2.37, *p* = 0.002) and methamphetamine and alcohol (mean difference = 2.25, *p* = 0.004), and a trend was observed for increased KSS scores between placebo and methamphetamine at this timepoint (mean difference = 1.69, *p* = .056; Figure 4).

#### Neurocognitive performance

A summary of neurocognitive outcomes is presented in Table 2, and the significant condition, time and interaction effects are reported in text later. Non-significant effects are not reported here for the sake of brevity.

**Table 2.** Mean scores (±SD) a linear fixed-effect model summary for neurocognitive task outcomes per treatment condition (placebo, alcohol, methamphetamine and methamphetamine and alcohol).^1^.

Outcome measure	Placebo			Alcohol			Methamphetamine			Methamphetamine and alcohol		
	Mean (SD)			Mean (SD)			Mean (SD)			Mean (SD)		
	Time 1	Time 2	Overall	Time 1	Time 2	Overall	Time 1	Time 2	Overall	Time 1	Time 2	Overall
Reaction time												
Simple reaction time (ms)	319.83 (60.50)	322.92 (69.52)	321.38 (64.13)	305.47 (49.33)	317.01 (66.05)	311.24 (57.58)	312.32 (65.50)	318.94 (68.85)	315.63 (66.19)	316.12 (49.87)	306.07 (48.54)	311.10 (48.67)
Complex reaction time (ms)	448.51 (86.07)	427.89 (74.92)	438.53 (80.21)	447.50 (87.38)	441.08 (74.10)	444.29 (79.67)	435.87 (78.36)	427.48 (70.71)	431.67^a*^ (73.54)	440.54 (89.89)	425.54 (74.11)	433.04^a*^ (81.40)
Complex accuracy (%)	96.38 (2.25)	96.00 (2.43)	96.19 (2.98)	94.40 (5.25)	94.67 (4.88)	94.53 (4.98)	96.75 (3.34)	97.12 (3.50)	96.94^a*^ (3.7)	94.50 (5.03)	96.25 (4.25)	95.38 (4.67)
Digit vigilance												
Reaction time (ms)	446.04 (49.85)	453.30 (59.36)	449.67 (54.05)	453.53 (51.55)	464.04 (64.41)	458.79^b*^ (57.67)	440.11 (55.09)	443.89 (61.84)	441.99^a**^ (57.64)	448.74 (52.41)	446.74 (53.55)	447.74^a*^ (52.13)
Accuracy (%)	94.80 (4.60)	94.88 (4.67)	97.43 (3.58)	95.17 (6.71)	93.46 (5.62)	94.89^b*^ (6.63)	95.39 (8.10)	91.64 (10.88)	96.87 (4.72)	95.56 (4.50)	93.71 (6.56)	97.63^a*^ (3.20)
False alarms	0.56 (1.09)	0.25 (0.45)	0.41 (0.84)	0.60 (0.91)	0.47 (9.64)	0.53 (0.78)	0.63 (0.96)	0.06ᶿ* (0.25)	0.34 (0.75)	0.56 (0.96)	0.38 (0.62)	0.47 (0.80)
Numeric working memory												
Reaction time (ms)	651.64 (144.40)	609.38 (120.22)	630.51 (132.31)	622.06 (164.45)	593.68 (148.39)	608.36 (154.77)	620.01 (112.62)	553.64ᶿ* (150.78)	587.27^b*^ (134.49)	592.16 (115.91)	580.39 (110.09)	586.27 (111.23)
Accuracy (%)	94.80 (4.60)	94.89 (4.57)	94.84 (4.56)	95.17 (6.71)	93.45 (5.62)	94.31 (6.14)	95.40 (8.10)	91.64 (10.88)	93.52 (9.63)	95.59 (4.50)	93.71 (6.56)	94.65 (5.61)
Spatial working memory												
Reaction time (ms)	607.34 (123.03)	611.87 (138.25)	635.78 (137.21)	564.80 (138.35)	560.97 (116.78)	599.14 (120.52)	591.19 (133.55)	587.88 (114.36)	617.18 (127.80)	578.88 (129.36)	566.97 (122.35)	590.22^b*^ (115.46)
Accuracy (%)	94.37 (5.06)	95.14 (5.19)	94.76 (5.06)	93.85 (4.91)	95.63 (3.29)	94.74 (4.21)	95.62 (4.51)	94.37 (4.65)	95.00 (4.55)	95.07 (3.47)	95.14 (4.65)	95.10 (3.44)

1 *N* = 16.

a Indicates significant difference in mean scores from alcohol, post-hoc paired t-test analyses with Bonferroni adjustment at *p *<* 0.05, or **p < 0.001.

b Indicates significant difference in mean scores from placebo, post-hoc paired t-test analyses with Bonferroni adjustment at *p *<* 0.05, or **p < 0.001.

ᶿ Indicates significant difference from Time 1 post-hoc paired t-test analyses with Bonferroni adjustment at *p *<* 0.05.

##### Simple and choice reaction time

There was a main effect of condition for complex reaction time accuracy (%) (F_(3, 103)_ = 3.79, *p* = 0.013) with poorer performance under the alcohol versus methamphetamine condition (mean difference = −2.29, *p* = 0.005). There was a main effect of time (F_(3, 102)_ = 7.73, *p* = 0.006) and condition (F_(3, 102)_ = 4.27, *p* = 0.007) for Complex reaction time (ms), with greater response time (poorer performance) in the alcohol versus methamphetamine and methamphetamine and alcohol conditions (mean difference = 18.31, *p* = 0.012; mean difference = 16.93, *p* = 0.024, respectively), and marginally faster reaction time at Time 2 compared to Time 1 (mean difference = −15.00, *p* = 0.054).

##### Digit vigilance

Bivariate analyses revealed an effect of sex on digit vigilance accuracy (%), with improved performance among female participants relative to males (mean difference = 1.75, *p* = 0.044). Controlling for sex, there was a significant main effect of condition for vigilance accuracy (%) (F_(3, 103)_ = 3.48, *p* = 0.019) with poorer performance (reduced accuracy) in the alcohol versus methamphetamine and alcohol condition (mean difference = −2.90 *p* *=* 0.027) and placebo condition (mean difference −2.70, *p* *=* 0.049). There was also a main effect of condition for vigilance reaction time (ms) (F_(3, 103)_ = 7.16, *p* < 0.001), with reduced performance (increased reaction time) in the alcohol condition relative to placebo (mean difference = 15.20, *p* = 0.022), methamphetamine (mean difference = 22.88, *p* < 0.001) and methamphetamine and alcohol conditions (mean difference = 17.13, *p* = 0.007). Bivariate analyses revealed an effect of sex on digit vigilance false alarms (%), with improved performance (fewer false alarms) reported by females relative to males (mean difference −0.54, *p* < 0.001). Controlling for sex, there was a significant main effect for time for vigilance false alarms (F_(3, 103)_ = 7.15, *p* < 0.009), with improved performance (fewer false alarms) in the methamphetamine condition at Time 2 versus Time 1 (mean difference = −0.56, *p* = 0.021) among females (*p* = 0.038).

##### Numeric working memory

There was a main effect of condition on numeric working memory reaction time (ms) (F_(3, 100)_ = 3.36 *p* = 0.022), with improved performance (lower reaction time) in the methamphetamine versus placebo condition (mean difference = −48.89, *p* = 0.016), as well as a main effect of time on numeric working memory reaction time (ms) (F_(1, 97)_ = 6.63 *p* = 0.012), with improved performance (faster reaction time) in the methamphetamine condition from Time 1 to Time 2 (mean difference = −63.53, *p* = 0.016).

##### Spatial working memory

Bivariate analyses revealed an effect of sex on spatial working memory reaction time (ms), with improved performance (faster responses) reported for female participants relative to males (mean difference −65.13, *p* = 0.005). Controlling for sex, there was a main effect of condition on spatial working memory reaction time (ms) (F_(3, 100)_ = 6.45 *p* < 0.001), with improved performance (faster reaction time) in the methamphetamine and alcohol versus placebo condition (mean difference = 53.17, *p* *<* 0.001).

## Discussion

Acute weighted oral doses of methamphetamine, when used alone or in combination with low doses of alcohol, produce prototypical physiological and subjective drug effects but do not reliably affect higher-order neurocognitive skills relative to alcohol-use alone. Sustained subjective stimulative effects and absence of additive performance deficits in the combined condition suggest at least some masking of biphasic alcohol effects over time, which may underlie motivations for co-consumption in recreational settings and increase propensity for harm.

Even at low doses (mean BAC 0.029%), alcohol impaired performance in tasks of psychomotor speed and sustained attention, but not working memory. Impairment in these functional domains supports low-effect thresholds for alcohol intoxication starting from a very low BAC (Dry et al., 2012) and underscores established implications for relevant safety-sensitive behaviours, including driving (Garrisson et al., 2021). Lack of clear effect of time-on-task performance despite clear subjective sedating-type effects of alcohol consumption may more closely reflect the constrained BAC peak and rapid elimination in biological matrices when used at these low doses, rather than a true lack of effect *per se*. While the average BAC was marginally lower than the target, it fell within the intended range, and evidence of significant objective and subjective outcomes (stimulant, sedative) confirm the presence of a biphasic (ascending and descending) alcohol concentration–effect curve (Jones, 2019).

Previous research has found that methamphetamine produced isolated improvement in psychomotor response speed during a working memory task and improved time-on-task performance proportionate to expected pharmacodynamic properties (Cruickshank and Dyer, 2009). Low-moderate doses of amphetamine-type stimulants improve select sustained attention and vigilance (Silber et al., 2006) and reduce psychomotor speed/response time (Narayan et al., 2021); however, some studies cite a lack of effect (Ermakova et al., 2014) or else note that these beneficial effects appear to dissipate when higher doses are consumed, particularly in tasks of complex higher-order functioning (inverted-U; Dolder et al., 2018; Naylor et al., 1985). Long-term or heavy patterns of use are also associated with enduring cognitive change, which may reflect alterations in brain morphology even in the absence of acute intoxication (Volkow et al., 2001). Considering the overrepresentation of methamphetamine-related harm, it is possible that more severe negative functional performance effects more closely reflect recreational patterns of consumption that are difficult to replicate experimentally (O’Malley et al., 2022). Recreationally, crystalline methamphetamine is typically consumed in binge-like patterns over a period of several hours or even days and is more commonly consumed via inhalation (smoking) or intravenously (injection) (McKetin et al., 2021). In its powdered form, it is more likely to be consumed intranasally (snorting). Additional work is therefore warranted to discern the effects of longer-term methamphetamine consumption to discern potential interactions of use patterns and safety-relevant indices.

Isolated impairment in the alcohol-only condition, relative to the combined methamphetamine and alcohol condition on measures of vigilance, and a general observation of preserved accuracy of performance without compromised speed of response may reflect ability of the psychostimulant to protect against gradual performance deterioration; however, these results were somewhat limited in scope. Nevertheless, the stimulant properties of methamphetamine were not altered by the biphasic effects of low doses of ethanol, and thus it is possible that there is at least some protective effect of methamphetamine against gradual performance degradation, even after lower doses of alcohol are consumed. The longevity of subjective effects and absence of clear sedation in the combined methamphetamine and alcohol condition, even beyond the expected half-life of alcohol and in contrast to known concentration curves, may increase the likelihood of repeated dosing (consuming more alcohol) for a longer duration. Indeed, methamphetamine pharmacokinetics were not altered by the concurrent administration of ethanol, which is consistent with previous acute dosing studies at similar doses (Mendelson et al., 1995). Amphetamine (including methamphetamine) and alcohol are often found in combination in cases of serious road trauma (Hayley et al., 2022), and polydrug use is associated with higher odds of risky road-user behaviour even in laboratory settings (such as running red light; Simons et al., 2012). From a signal detection perspective, we show greater ratio of false alarms/misses on the vigilance task following alcohol usage, but not in the combined use condition. More work is needed to explore how co-use of these substances may translate to these types of operational errors during driving. It is possible that reduced feelings of intoxication, coupled with similar drug bioavailability and metabolism, contribute to real and sustained risk due to incongruence between perceived and actual effects, which influence these outcomes.

The small sample size is a notable limitation of the present study; however, the closely controlled design, coupled with multiple testing timepoints per experimental session for each treatment condition, ensures high data quality and expands a very limited research area. To date, only two studies have implemented a comparable combined methamphetamine and alcohol-dosing paradigm (Mendelson et al., 1995; Kirkpatrick et al., 2012a), each having yielded a much smaller evaluable sample than examined here. We overcame some key limitations of previous research by ensuring a non-smoking paradigm (Kirkpatrick et al., 2012a) and by mirroring weighted doses of treatment(s) used in previous behavioural studies of methamphetamine (Silber et al., 2006). As an acute dosing paradigm was used, we are unable to comment on the potential interactive effects of these substances as they may be replicated under repeated and/or chronic dosing schedules or following delayed intake. As treatments were consumed simultaneously, we recognise that we are unable to generalise to a more naturalistic environment whereby individuals might consume alcohol at a later stage (i.e. 1–2 h after) to counteract the more negative stimulative properties of methamphetamine. Such laboratory research would provide valuable insight into drug effects under the most precise pharmacological conditions possible; however, this approach would need to be considered in terms of what is methodologically and ethically acceptable. Given known sex differences in the physiological response to both psychostimulants and alcohol (Baraona et al., 2001; Franconi et al., 2007), and evidence of select sex-related cognitive effects found in this study, future research should also include sex as a primary factor to determine the magnitude of main effects and potential interactions. Subjective drug effects (B-BAES) were compared to placebo and were not evaluated at baseline. This precludes examination of condition-specific differences under these conditions, and we are unable to assess change from baseline. Notwithstanding these limitations, the present results provide the most robust examination on the neurocognitive effects of methamphetamine alone, and in combination with alcohol to date.

Despite reductions in the global consumption of alcohol, rates of methamphetamine used continue to increase. Polydrug use represents an increasing concern for safety, and discerning potential interactive effects on performance and behaviour is critical for informing effective harm reduction strategies. Dissociation between these subjective effects and objective effects appears partially supported, whereby the stimulative effects of methamphetamine may mask some of the biphasic sedative properties of even low doses of alcohol. In practical terms, this may potentiate the reinforcing and hazardous effects of each drug and increasing the propensity for harm.

## Supplemental Material

sj-docx-1-jop-10.1177_02698811231179805 – Supplemental material for Acute neurocognitive and subjective effects of oral methamphetamine with low doses of alcohol: A randomised controlled trial Click here for additional data file. Supplemental material, sj-docx-1-jop-10.1177_02698811231179805 for Acute neurocognitive and subjective effects of oral methamphetamine with low doses of alcohol: A randomised controlled trial by Amie C Hayley, Brook Shiferaw, Joanna Rositano and Luke A Downey in Journal of Psychopharmacology

## References

[bibr1-02698811231179805] Åkerstedt T Gillberg M (1990) Subjective and objective sleepiness in the active individual. Int J Neurosci 52: 29–37. 2265922 10.3109/00207459008994241

[bibr2-02698811231179805] Cruickshank CC Dyer KR (2009) A review of the clinical pharmacology of methamphetamine. Addiction 104: 1085–1099. 19426289 10.1111/j.1360-0443.2009.02564.x

[bibr3-02698811231179805] Baraona E Abittan CS Dohman K , et al. (2001) Gender differences in pharmacokinetics of alcohol. Alcohol Clin Exp Res 25: 502–507. 11329488

[bibr4-02698811231179805] Dolder PC Strajhar P Vizeli P , et al. (2018) Acute effects of lisdexamfetamine and D-amphetamine on social cognition and cognitive performance in a placebo-controlled study in healthy subjects. Psychopharmacology 235: 1389–1402. 29511807 10.1007/s00213-018-4849-0

[bibr5-02698811231179805] Dry MJ Burns NR Nettelbeck T , et al. (2012) Dose-related effects of alcohol on cognitive functioning. PLOS One 7: e50977. 10.1371/journal.pone.0050977 PMC3510176 23209840

[bibr6-02698811231179805] Ermakova AO Ramachandra P Corlett PR , et al. (2014) Effects of methamphetamine administration on information gathering during probabilistic reasoning in healthy humans. PLoS One 9(7): e102683. 10.1371/journal.pone.0102683 PMC4111474 25061949

[bibr7-02698811231179805] Garrisson H Scholey A Ogden E , et al. (2021) The effects of alcohol intoxication on cognitive functions critical for driving: A systematic review. Accid Anal Prev 154: 106052. 33676142 10.1016/j.aap.2021.106052

[bibr8-02698811231179805] Franconi F Brunelleschi S Steardo L , et al. (2007). Gender differences in drug responses. Pharmacol Res 55: 81–95. 17129734 10.1016/j.phrs.2006.11.001

[bibr9-02698811231179805] Hayley AC Ogeil RP Faulkner A , et al. (2022) The incidence and temporal patterns of use of amphetamine-type stimulant use in traffic-related ambulance attendances from 2015 to 2020 in Victoria, Australia. J Stud Alcohol Drugs 84: 128–136. 10.15288/jsad.22-00050 36799683

[bibr10-02698811231179805] Isoardi KZ Ayles SF Harris K , et al. (2019) Methamphetamine presentations to an emergency department: Management and complications. Emerg Med Australas 31: 593–599. 30592564 10.1111/1742-6723.13219

[bibr11-02698811231179805] Jones AW (2019) Alcohol, its absorption, distribution, metabolism, and excretion in the body and pharmacokinetic calculations. Wiley Interdiscip Rev: Forensic Sci 1: e1340.

[bibr12-02698811231179805] Kirkpatrick MG Gunderson EW Levin FR , et al. (2012a) Acute and residual interactive effects of repeated administrations of oral methamphetamine and alcohol in humans. Psychopharmacology 219: 191–204. 21748253 10.1007/s00213-011-2390-5 PMC3220757

[bibr13-02698811231179805] Kirkpatrick MG Gunderson EW Perez AY , et al. (2012b) A direct comparison of the behavioral and physiological effects of methamphetamine and 3,4-methylenedioxymethamphetamine (MDMA) in humans. Psychopharmacology 219: 109–122. 21713605 10.1007/s00213-011-2383-4 PMC4430833

[bibr14-02698811231179805] Martin CS Earleywine M Musty RE , et al. (1993) Development and validation of the biphasic alcohol effects scale. Alcohol Clin Exp Res 17: 140–146. 8452195 10.1111/j.1530-0277.1993.tb00739.x

[bibr15-02698811231179805] McKetin R Sutherland R Peacock A , et al. (2021) Patterns of smoking and injecting methamphetamine and their association with health and social outcomes. Drug Alcohol Rev 40: 1256–1265. 34365687 10.1111/dar.13364 PMC9292494

[bibr16-02698811231179805] Mendelson J Jones RT Upton R , et al. (1995) Methamphetamine and ethanol interactions in humans. Clin Pharmacol Ther 57: 559–568. 7768079 10.1016/0009-9236(95)90041-1

[bibr17-02698811231179805] Narayan AJ Aitken B Downey LA , et al. (2021) The effects of amphetamines alone and in combination with alcohol on functional neurocognition: A systematic review. Neurosci Biobehav Rev 131: 865–881. 34626687 10.1016/j.neubiorev.2021.10.003

[bibr18-02698811231179805] Naylor H Halliday R Callaway E (1985) The effect of methylphenidate on information processing. Psychopharmacology 86: 90–95. 3927371 10.1007/BF00431690

[bibr19-02698811231179805] Volkow ND Chang L , et al. (2001) Association of dopamine transporter reduction with psychomotor impairment in methamphetamine abusers. Am J Psychiatry 158: 377–382. 11229977 10.1176/appi.ajp.158.3.377

[bibr20-02698811231179805] O’Malley KY Hart CL Casey S , et al. (2022) Methamphetamine, amphetamine, and aggression in humans: A systematic review of drug administration studies. Neurosci Biobehav Rev 141: 104805. 35926727 10.1016/j.neubiorev.2022.104805

[bibr21-02698811231179805] Rositano J Harpas P Kostakis C , et al. (2016) Supported liquid extraction (SLE) for the analysis of methylamphetamine, methylenedioxymethylamphetamine and delta-9-tetrahydrocannabinol in oral fluid and blood of drivers. Forensic Sci Int 265: 125–130. 26878366 10.1016/j.forsciint.2016.01.017

[bibr22-02698811231179805] Rueger SY King AC (2013) Validation of the brief biphasic alcohol effects scale (B-BAES). Alcohol: Clin Exp Res 37: 470–476. 23078583 10.1111/j.1530-0277.2012.01941.x PMC3570663

[bibr23-02698811231179805] Silber BY Croft RJ Papafotiou K , et al. (2006) The acute effects of d-amphetamine and methamphetamine on attention and psychomotor performance. Psychopharmacology 187: 154–169. 16761129 10.1007/s00213-006-0410-7

[bibr24-02698811231179805] Simons R Martens M Ramaekers J , et al. (2012) Effects of dexamphetamine with and without alcohol on simulated driving. Psychopharmacology 222: 391–399. 22076246 10.1007/s00213-011-2549-0 PMC3395339

[bibr25-02698811231179805] Singh AK (2019) Alcohol interaction with cocaine, methamphetamine, opioids, nicotine, cannabis, and γ-Hydroxybutyric acid. Biomedicines 7: 16. 30866524 10.3390/biomedicines7010016 PMC6466217

[bibr26-02698811231179805] United Nations (2020) World Drug Report 2020. The United Nations Office on Drugs and Crime (UNODC).

[bibr27-02698811231179805] Vinarov Z Abdallah M Agundez JAG , et al. (2021) Impact of gastrointestinal tract variability on oral drug absorption and pharmacokinetics: An UNGAP review. Eur J Pharm Sci 162: 105812. 33753215 10.1016/j.ejps.2021.105812

[bibr28-02698811231179805] Wang L Min JE Krebs E , et al. (2017) Polydrug use and its association with drug treatment outcomes among primary heroin, methamphetamine, and cocaine users. Int J Drug Policy 49: 32–40. 28888099 10.1016/j.drugpo.2017.07.009 PMC5681890

[bibr29-02698811231179805] Wesnes KA Brooker H Ballard C , et al. (2017) Utility, reliability, sensitivity and validity of an online test system designed to monitor changes in cognitive function in clinical trials. Int J Geriatr Psychiatry 32: e83–e92. 10.1002/gps.4659 28128869

[bibr30-02698811231179805] Wu L-T Pilowsky DJ Schlenger WE , et al. (2007) Misuse of methamphetamine and prescription stimulants among youths and young adults in the community. Drug Alcohol Depend 89: 195–205. 17257780 10.1016/j.drugalcdep.2006.12.020 PMC2063507

