# Supplementary material for: Acute neurocognitive and subjective effects of oral methamphetamine with low doses of alcohol: A randomised controlled trial
Source: J Psychopharmacol. 2023 Jun 14;37(9):928–36. doi: 10.1177/02698811231179805 (PMC10481625; doi:10.1177/02698811231179805)
Supplement: sj-docx-1-jop-10.1177_02698811231179805 – Supplemental material for Acute neurocognitive and subjective effects of oral methamphetamine with low doses of alcohol: A randomised controlled trial [file sj-docx-1-jop-10.1177_02698811231179805.doc]

**
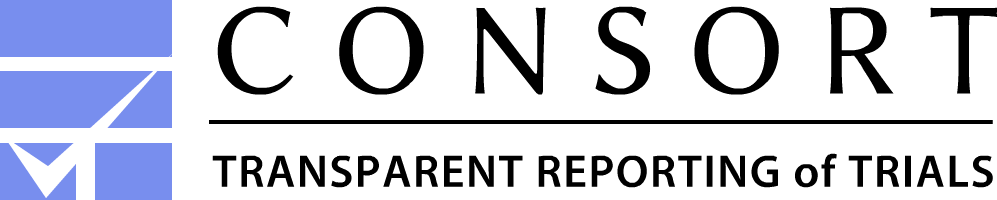
**

**CONSORT 2010 Flow Diagram**

**Allocation**

**Analysis**

**Enrollment**

Assessed for eligibility (n= 70)

Excluded (n=29)

♦  Not meeting inclusion criteria (n= 11 )

♦  Declined to participate (n= 1)

♦  Lost to follow up (n= 16)

♦  Withdrew (n= 1)

Discontinued intervention (n= 9)

- Withdrew (n = 6)

* Completed 3 session (n = 2)

* Completed 2 sessions (n = 2)

* Completed 1 session (n = 2)

- Lost to follow up (COVID-19) (n = 3)

* Completed 3 sessions (n = 1)

* Completed 2 sessions (n = 1)

* Completed 1 session (n = 1)

Analysed (n= 16)
♦ Excluded from analysis (n= 5)

* Data loss due to technical failure (n= 4)

* Protocol violation (n = 1)

Randomized (n= 41)

Allocated to intervention (n= 41)

♦ Received allocated intervention (n= 30)

♦ Did not receive allocated intervention (give reasons) (n= 11)

- Lost to follow up (general) (n = 8)

- Lost to follow up (COVID-19) (n = 3)
